# Dose fall-off during the treatment of thoracic spine metastasis with CyberKnife stereotactic body radiation therapy (SBRT)

**DOI:** 10.17305/bjbms.2018.3185

**Published:** 2020-02

**Authors:** Zhongjian Ju, Jingyuan Wang, Huaiwen Zhang, Lei Du, Wei Xu, Xiaoshen Wang, Ruigang Ge, Jiwei Li, Qingzeng Zheng, Jianxiong Li

**Affiliations:** 1 Department of Radiation Oncology, Hainan Branch of Chinese PLA General Hospital, Sanya, China; 2 Department of Radiotherapy, Chinese PLA General Hospital, Beijing, China; 3 Department of Radiotherapy, Jiang-Xi Cancer Hospital, Nanchang, China; 4 Department of Radiotherapy, Beijing Geriatric Hospital, Beijing, China

**Keywords:** CyberKnife, dosage, radiation, stereotactic body radiation therapy, SBRT, thoracic spine metastasis, dose gradient

## Abstract

CyberKnife stereotactic body radiation therapy (SBRT) is becoming increasingly used for cancer treatment and, to maximize its clinical application, it is important to define the dosimetric characteristics, optimal dose, and fractionation regimens. The aim of this study was to evaluate the dose fall-off in two fractionated regimens of CyberKnife SBRT during the treatment of thoracic spinal metastasis. Patients with spinal metastasis involving a vertebra and pedicle were treated with 40 Gy in 5 fractions (n = 4), and patients with spinal metastasis involving only a vertebra received 33 Gy in 3 fractions (n = 4). A new approach was used to measure absolute dose fall-off distance, relative dose fall-off distance, and the dose fall-off per unit distance along four reference directions in the axial plane. Patients treated with 33 Gy/3 fractions had a greater absolute dose fall-off distance in direction 1 (from the point with maximum dose [Dmax] towards the spinal cord) and direction 3 (the opposite of direction 1), a greater relative dose fall-off distance in direction 3, and a lower dose fall-off per unit distance in direction 1 and 3 compared to patients treated with 40 Gy/5 fractions (all *p* < 0.05). Overall, the dose fall-off towards the spinal cord is rapid during the treatment of thoracic spinal metastasis with CyberKnife SBRT, which allows a higher dose of radiation to be delivered to the tumor and, at the same time, better protection of the spinal cord.

## INTRODUCTION

Bone metastases can occur in up to 48% of patients with stage IV lung cancer [1,2], as well as in patients with other common primary solid tumors, such as breast cancer, prostate cancer [3] and renal carcinoma [4]. Symptomatic spinal metastases may develop in up to 10% of cancer patients [5]. Bone metastases often lead to increased bone resorption, which may cause fractures, spinal cord compression, and severe bone and neuropathic pain [5-7]. Metastatic spinal cord compression is a common complication of cancer, with back pain being a common symptom, and can present as an oncologic emergency [8].

Conventional radiotherapy, delivered in 5 to 20 daily fractionations, can provide pain relief in approximately 70% to 80% of patients with spinal metastasis within three months [9,10]. However, it is not used to treat recurrent spinal metastases. Stereotactic body radiation therapy (SBRT) has become a recognized treatment for spinal metastases, providing rapid pain relief (i.e., within 24–72 hours) in 84% to 90% of patients that lasts for 1 year or longer [4,5,10-12]. It is especially beneficial for patients who are not candidates for surgical therapy [4]. The CyberKnife system (Accuracy, Inc., Sunnyvale, CA) is a stereotactic radiosurgery platform that provides real-time image tracking of internal anatomical structures (e.g., the skull). This enables monitoring of the position of lesion, alignment of the independent beams to treatment target, and relatively precise delivery of radiation of 100 to 150 independent beams at a prespecified distance, to avoid or reduce exposure of radiosensitive organs, such as the spinal cord [10]. Its robotic arm has 6 axes of freedom, and is coupled with two ceiling-mounted X-ray cameras for monitoring target position [10]. The CyberKnife system has a unique Xsight spine tracking mode, which allows real-time tracking of changes in the target area of the spine and the control of position error within a range of 0.53 ± 0.16 mm during treatment [13]. The CyberKnife system provides better pain control compared to routine radiotherapy, and thereby provides a more precise and safer treatment [5,10,13]. Dosimetric characterization of the CyberKnife for spinal metastases typically ranges from 8 Gy to 24 Gy for a single dose, and from 18 Gy to 36 Gy for multiple fractions [14]. The dose fall-off gradient can be used for evaluating the quality of treatment plan and selecting different treatment methods [15].

We hypothesized that the dose administered with the CyberKnife and/or the primary direction of beams may affect the dose fall-off variables. Thus, the objective of this study was to determine the relationship between the primary directions of beams towards the target area in the CyberKnife system and the tendency of dose fall-off along the different directions.

## MATERIALS AND METHODS

### Patients

Patients with a single spinal metastasis, who were admitted to our hospital between November 2011 and August 2013 and were expected to survive more than 6 months, were recruited to this study. The inclusion criteria were a Karnofsky Performance Status (KPS) score greater than 65 and Tumor, Node, Metastasis (TNM) stage IV at the time of diagnosis. Patients in whom bone metastases were found after surgery were excluded.

This study was approved by the Institutional Review Board of our hospital, and all patients provided written informed consent.

### Scanning and definition of the target area and important organs

The patient was placed in the supine position and fixed with vacuum pads. Computed tomography (CT) locating was performed using the Philips Brilliance CT Big Bore CT scanner (Netherlands), and the slice thickness was 1.5 mm. The obtained images were transferred to the MultiPlan 4.0.2 system via DICOM to outline the target area and organs at risk.

The medical physicists outlined the target area and organs at risk on the CT images, as previously described [16]. The target areas included gross tumor volume (GTV) and planned target volume (PTV). GTV was identified by extrapolating images from magnetic resonance imaging (MRI) or positron emission tomography (PET)-CT, and outlining the tumor area on the CT images. The PTV was defined with 3 mm margins beyond the GTV in all dimensions. Because the metastases of all patients were located in the thoracic region, the organ at risk was the region of spinal cord exposed on the CT bone window. Radiation myelopathy rarely occurs, and thus the musculature is not considered an organ at risk.

Maximum dose (D_max_) was considered the center, and was calculated as the highest point by the planning system for each patient. The 70% isodose line, instead of the median (80%) isodose line (70–94%), was used to cover the PTV in accordance with the characteristics of the CyberKnife system.

### Treatment plan design and dosing

**FIGURE 1:**
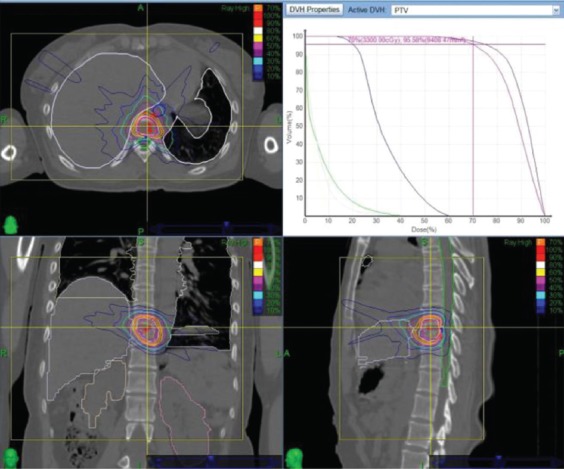
Dose distribution of the treatment plan and dose-volume histogram.

The target area and organs at risk were outlined using the MultiPlan 4.0.2 in the CyberKnife system, and a SBRT plan was designed for each patient. The SBRT used two collimators and the dose rate was 800 cGy/min. The extent of target area coverage was increased as much as possible, while the dose limits for normal organs were maintained. Each treatment plan guaranteed that 70% of the isodose line covered more than 85% of the target volume. The dose distribution is shown in Figure 1.

The collimator size was selected according to the target volume and clinical experience, and was thus different in each patient. In general, a collimator size of 1/3 and 2/3 of maximal diameter will achieve the best results, which is also recommended by the CyberKnife system.

A dose of 40 Gy was administered in five fractions to the PTV of patients with vertebral and pedicle metastasis. These patients had primary gastric, lung, or rectal cancer; KPS scores of 65 to 75; and a GTV of <5 mm to the spinal cord. The dose limit for the spinal cord in the five-fraction treatment mode was: D_max_ < 30 Gy, V_22.5_ < 0.25 ml, and V_13.5_ < 1.2 ml [17].

A dose of 33 Gy was given in three fractions to the PTV of patients with a single vertebral metastasis. These patients had primary lung or nasopharyngeal cancer, and a distance of <5 mm between the GTV and spinal cord. Dose limit for the spinal cord in the three-fraction treatment mode was D_max_ < 22 Gy, V_18_ < 0.25 ml, and V_11.1_ < 1.2 ml [17].

A number of methods were used to assure target coverage and decrease of radiation dose to the spinal cord: several cold help regions were defined in the target area to improve the coverage; 8 shells with different diameters and different dose limitations were defined around the tumor to control the low-dose area; 3–5 hot help structures were defined in the spinal cord area to reduce the dose received by the spinal cord.

### Measurement of parameters related to the dose gradient

**FIGURE 2:**
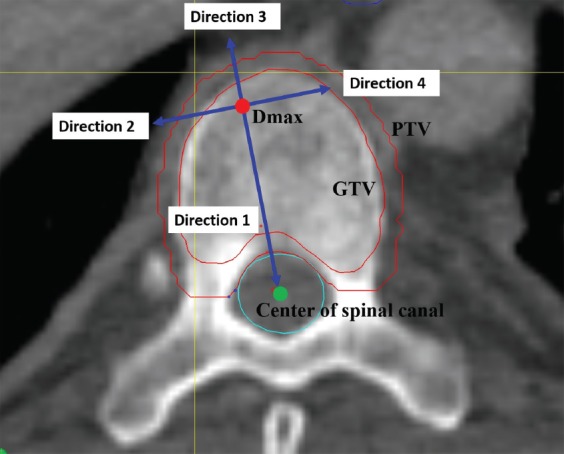
Diagram of the reference directions. The red point denotes the D_max_. The green point denotes the center of the spinal canal. The outer red line denotes the contour of the PTV. The inner red line denotes the contour of the GTV. The green line denotes the contour of the spinal canal. The blue arrows denote the direction 1, 2, 3, and 4. D_max_ Maximum dose; GTV: Gross tumor volume; PTV: Planned target volume.

The point of maximum dose was taken as the center, and the line connecting it with the spinal cord center was considered to be the direction of the spinal cord (direction 1; Figure 2). The other three directions, evenly spaced 90° apart, were designated as direction 2, 3, and 4 (Figure 2).

**FIGURE 3:**
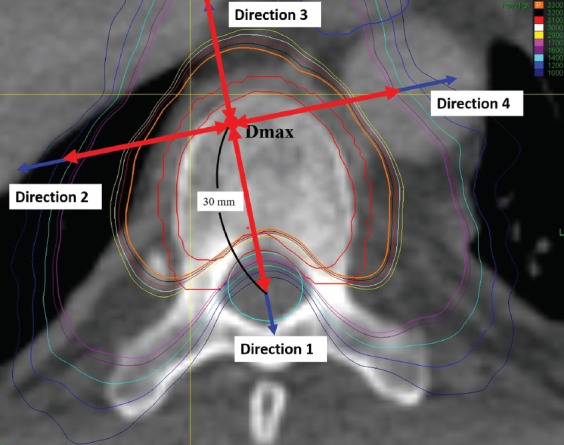
Measurement of absolute dose fall-of distance. The double-ended red arrows indicate the range of measurements. The red point denotes the D_max_. The blue arrows denote the direction 1, 2, 3 and 4. D_max_ Maximum dose.

**FIGURE 4:**
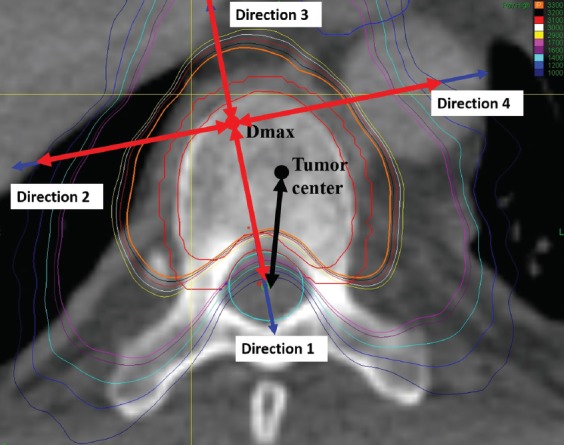
Measurement of relative dose fall-off distance. The double-ended red arrows indicate the range of measurements. The red point denotes the D_max_. The blue arrows denote the direction 1, 2, 3 and 4. The black point denotes the center of tumor. The black arrow denotes the direction from the center of tumor to the center of the spinal canal. D_max_: Maximum dose.

**FIGURE 5:**
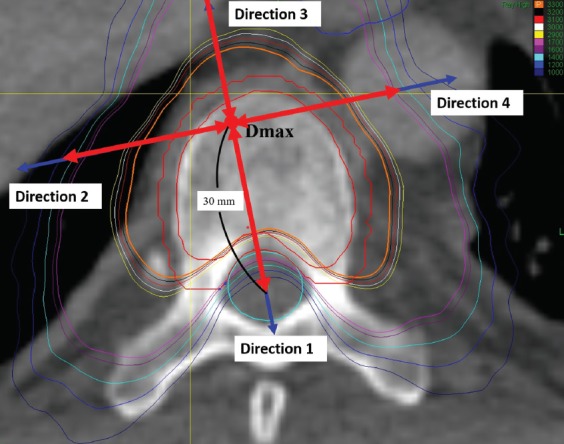
Measurement of dose fall-off per unit distance. The double-ended red arrows indicate the range of measurements. The red point denotes the D_max_. The blue arrows denote the direction 1, 2, 3 and 4. D_max_ Maximum dose.

For measurement of absolute dose fall-off, the distance between two adjacent points of dose fall-off of 100 cGy was measured between the starting point of prescribed dose and the point of its 30% dose (Figure 3). The relative dose fall-off distance was the distance between two adjacent points of dose fall-off 5% from the starting point of maximum dose to the point of 30% dose (Figure 4). For measurement of the dose fall-off per unit distance, the changes in dose were measured every 1 mm from the starting point extending outwards from the starting point of maximum dose to a point 30 mm away (Figure 5).

### Statistical analysis

Gender was reported as number (%). All continuous variables were expressed as mean ± standard deviation. Nonparametric statistical analysis was used in this study. Post-hoc pairwise comparison was performed using Wilcoxon signed-rank test with a significance level of 0.05. The Friedman test was used to compare differences among different directions in the two treatment groups. Treatment differences were compared in different directions using the Mann-Whitney U test. All statistical tests were 2-sided, and evaluated at the 0.05 level of significance. Statistical analyses were performed by IBM SPSS statistical software version 22 for Windows (IBM Corp., Armonk, New York, USA).

## RESULTS

A total of 8 patients (6 males and 2 females) with metastases in the thoracic spine and a mean age of 58.4 years were included in the study. Patients’ demographic data, health status, and location of thoracic spine metastasis are shown in Table 1 and S1. Five patients had primary lung carcinoma, and the other 3 had nasopharyngeal carcinoma, gastric adenocarcinoma or rectal adenocarcinoma. The thoracic spine metastases were at the first thoracic vertebra (T1) in 2 patients, T5 in 1, T9 in 2, T10 in 2, and T11 in 1 patient.

**TABLE 1:**
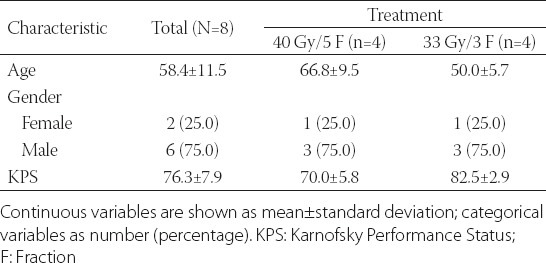
Patient characteristics

The single metastasis in 4 patients extended into the vertebra and the pedicle (patients treated with 40 Gy/5 fractions). The PTV had a median volume of 22.99 cc (range 4.58–28.61 cc), a median isodose value of 69% (range 65–70%), and median coverage of 91.95% (range 74.61–94.9%).

In 4 patients, the single metastasis involved only the vertebra (patients treated with 33 Gy/3 fractions). The PTV had a median volume of 12.42 cc (range 7.4–27.3 cc), a median isodose value of 70% (range 70–80%), and median coverage of 90.52% (range 88.6–96.2%).

### Comparison of the dose fall-off parameters in the target area

#### Absolute dose fall-off rate

The patients treated with 40 Gy/5 fractions had an average dose fall-off rate from the point of the prescribed dose (tumor margin) to the specified spinal cord dose (D_max_ < 3000 cGy) of 0.175 mm/100 cGy. The average dose fall-off rates in the four directions were significantly different [0.208, 0.720, 0.661, and 0.811 mm/100 cGy; *p* < 0.001] (Table 2). For example, the absolute dose fall-off rate in directions 2, 3, and 4 was significantly lower than in direction 1 (all *p* < 0.05). The absolute dose fall-off rate in direction 4 was significantly lower than in directions 2 and 3 (both *p* < 0.05), but that in direction 3 was significantly higher than that in direction 2 (*p* < 0.05).

**TABLE 2:**
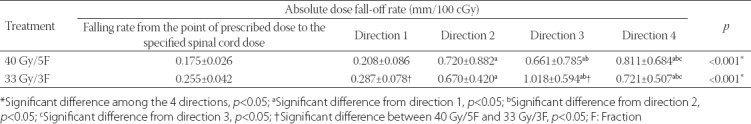
Absolute dose fall-off rate

The patients treated with 33 Gy/3 fractions had an average dose fall-off rate from the point of prescribed dose to the specified spinal cord dose (D_max_ < 2200 cGy) of 0.255 mm/100 cGy. The average dose fall-off rates in the four directions were significantly different [0.287, 0.670, 1.018, and 0.721 mm/100 cGy; *p* < 0.001] (Table 2). The average dose fall-off rate in directions 2, 3, and 4 was significantly lower than in direction 1 (all *p* < 0.05). The average dose fall-off rate in directions 3 and 4 was significantly lower than in direction 2 (both *p* < 0.05), but the dose fall-off rate in direction 4 was significantly higher than that in direction 3 (*p* < 0.05). In addition, the absolute dose fall-off rates in directions 1 and 3 in patients treated with 33 Gy/3 fractions were significantly lower than in patients treated with 40 Gy/5 fractions (both *p* < 0.05).

#### Relative dose fall-off distance

In patients treated with 40 Gy/5 fractions, there were significant differences in the relative dose fall-off distance in directions 1, 2, 3, and 4 (0.868, 1.477, 1.565, and 1.699 mm/5% D_max_, respectively; *p* < 0.001; Table 3). The relative dose fall-off distances in directions 2, 3, and 4 were significantly greater than in direction 1 (all *p* < 0.05), and the relative dose fall-off distance in direction 4 was significantly greater than in direction 3 (*p* < 0.05).

**TABLE 3:**
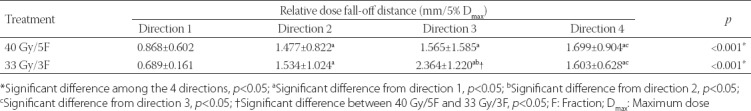
Relative dose fall-off distance

In patients treated with 33 Gy/3 fractions, there were significant differences in the relative dose fall-off distance in directions 1, 2, 3, and 4 (0.689, 1.534, 2.364, and 1.603 mm/5 % D_max_, respectively; *p* < 0.001; Table 3). The relative dose fall-off distances in directions 2, 3, and 4 were significantly greater than in direction 1 (all *p* < 0.05); and it was significantly greater in direction 3 than in direction 2 (*p* < 0.05). However, the relative dose fall-off distance in direction 4 was significantly lower than in direction 3 (*p* < 0.05). In addition, the relative dose fall-off distance in direction 3 was significantly greater in patients treated with 33 Gy/3 fractions compared to patients treated with 40 Gy/5fractions (*p* < 0.05).

#### Dose fall-off per unit distance

In patients treated with 40 Gy/5fractions, there were significant differences in the dose fall-off per unit distance in all four directions (353.628, 221.955, 243.669, and 196.428 cGy/mm; *p* < 0.001; Table 4). The dose fall-off per unit distance in directions 2, 3, and 4 were significantly lower than in direction 1 (all *p* < 0.05). The dose fall-off per unit distance in direction 3 was significantly higher than in direction 2 (*p* < 0.05) and direction 4 (*p* < 0.05).

**TABLE 4:**
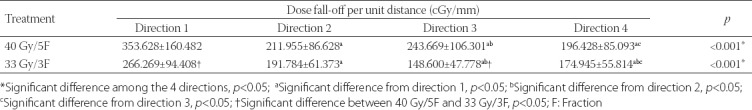
Dose fall-off per unit distance

In patients treated with 33 Gy/3 fractions, there were significant differences in the dose fall-off per unit distance in all four directions (266.269, 191.784, 148.6, and 174.945 cGy/mm; *p* < 0.001; Table 4). The dose fall-off per unit distance in directions 2, 3 and 4 was significantly lower than in direction 1 (all *p* < 0.05). The dose fall-off per unit distance was the highest in direction 1, followed by direction 2, direction 4 and direction 3 (*p* < 0.05). In addition, the patients receiving 40 Gy/5 fractions had significantly higher dose fall-off per unit distance in directions 1 and 3 than the patients treated with 33 Gy/3 fractions (both *p* < 0.05).

## DISCUSSION

The main findings of this study on CyberKnife treatment of spinal cord metastasis are that patients treated with a dose of 33 Gy in 3 fractions have lower absolute dose fall-off in directions 1 and 3 compared to patients treated with a dose of 40 Gy in 5 fractions, as well as a sharper and faster relative dose fall-off in directions 1 and 3 and a lower dose fall-off per unit distance. Both dosing patterns (i.e., 8 Gy × 5 and 11 Gy × 3) are commonly used in clinical practice. The biological equivalent dose (BED) was similar between the two patterns (BED_40_ Gy × 5 = 72 Gy; BED_33_ Gy × 3 = 69.3 Gy), and the efficacy was also comparable. In designing the treatment plans, the dosing pattern and spinal cord limitations were different; both were difficult to determine, although the dosing pattern was more difficult. Thus, the use of both patterns is clinically relevant, as both may provide evidence for dose selection in the future. In addition, our results showed differences in the dose-fall between the two dosing patterns.

Heron et al. found that both single session and multisession CyberKnife treatments to spinal metastasis provided effective pain relief for up to 1 year and local tumor control up to 2 years [18]. In another study, multivariate analysis showed that spinal cord compression and Eastern Cooperative Oncology Group (ECOG) performance status were significantly associated with pain relapse [7]. Nieder et al. showed that about half of the patients receiving SBRT for palliative treatment of spinal or brain metastases had a primary lung cancer [19], which is similar to our patient population. In the current study, we investigated whether the primary direction of the CyberKnife treatment affected the rate of dose fall-off, which could in turn affect the dose delivered to the spinal column.

The results of our study indicated that the absolute dose fall-off rate was significantly different among the four directions examined, and was significantly different between the two groups in directions 1 and 3. Although Lee et al. did not examine the dose fall-off in different directions, they reported that the homogeneity ratio of CyberKnife treatment plans in 2 patients with thoracic spinal metastasis was 1.23 and 1.25 [7]. In comparison, Hossain et al. reported that the average percentage of dose fall-off for the treatment of prostate cancer was 2.9 ± 0.8%/mm in the anterior direction, 3.8 ± 1.6%/mm in the posterior direction, and 3.6 ± 0.4%/mm in all directions [20]. Our results showed that the dose fall-off was relatively rapid in the direction of the spinal cord, according to the parameters set in the development of treatment plan. Of note, the CyberKnife Xsight Spine Tracking System corresponds to 81 associated locating points via the point of interest during DRR and real-time imaging, carries out non-rigid image registration, and realizes real-time tracking of the target area to achieve a treatment error of less than 0.53 mm [13].

In the present study, we found that the dose fall-off rate in the direction of the spinal cord was significantly steeper in the patients treated with a dose of 40 Gy than in the patients treated with a dose of 33 Gy. A possible reason is that the target area in the patients treated with 33 Gy included only the vertebral body, and it was easy to reach the spinal cord dose limit. In comparison, the patients treated with 40 Gy included the vertebral body together with the pedicle. Therefore, in the patients treated with 33 Gy, the dose distribution interval was relatively loose and the dose fall-off gradient was relatively slow.

The four directions selected in this study for dose fall-off investigation are not as common as anterior to the target volume, posterior to the target volume, and others. We selected appropriate directions for the investigation of dose fall-off after careful consideration. A few studies have investigated the dose fall-off in SBRT, and the previous methods used for intensity-modulated radiation therapy (IMRT) are not applicable. Thus, in this study we applied the newer standards. D_max_ was used only for the selection of direction and not as the starting point for the dose fall-off analyses. We studied the region spanning from the prescribed dose point to the 30% dose line. The prescribed dose basically covered the PTV. The spinal cord should be protected and, because it is close to the target lesion, the dose must be strictly controlled. The distance between different isodose lines and D_max_ reflects the actual dose fall-off. If other points are used (such as tumor center), there might be a bias. As shown in Figure 3 (black arrow), the direction from D_max_ to the spinal cord center is vertical to the isodose line. If the direction is selected from the tumor center, the distance is shortened and the dose fall-off rate increases, which is incorrect. We believe that the distance between D_max_ and isodose lines may better reflect the dose fall-off. With other radiation techniques, such as three-dimensional conformal radiotherapy (3DCRT) and IMRT, the PTV is usually at some distance from normal tissues. For example, in lung cancer the distance between the PTV and the spinal cord is typically larger than 5 cm, and thus the selection of direction may not significantly bias the results. In the present study, the PTV was close to the spinal cord, and a difference in the starting point of even 1 mm only will significantly impact the outcome. Another study has also calculated the conformity index using D_max_ [21].

There are a number of limitations to this study. First, the study included only 8 patients, and two different doses were used. Second, the selection of the center measurement point did not perfectly reflect the intent of an individual plan because the pathway from the maximum dose point to the spinal cord center might not be the shortest route. Third, because the prescribed dose in the tumor was normalized to 70% during the treatment plan design, some data inside the target area had to be discarded during statistical analysis. Lastly, we did not compare CyberKnife dose fall-off with other devices for radiation delivery.

## CONCLUSION

The Cyberknife provides rapid dose fall-off towards the spinal cord during the treatment of spine metastasis, which may protect the spinal cord from radiation-induced injury. The dose fall-off was different among the four directions, as well as between the patients treated with 40 Gy/5 fractions and those treated with 33 Gy/3 fractions, suggesting that optimal target area conformation plays a very important role in the protection of organs at risk.

## Figures and Tables

**TABLE S1:**
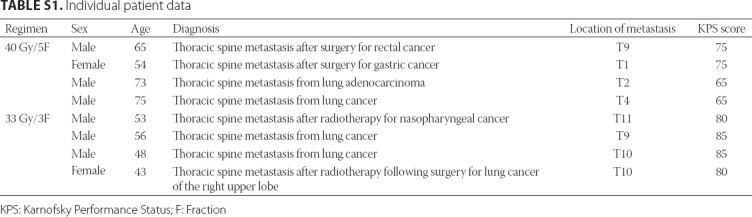
Individual patient data
